# Endovascular Treatment of Mirror Aneurysms in Subarachnoid Hemorrhage Patients: Single Stage or Multiple Stage?

**DOI:** 10.1002/brb3.70234

**Published:** 2024-12-31

**Authors:** Yunfei Song, Guoqiang Song, Guijing Liu, Limei Mao, Xiuhu An, Chao Peng, Jian Li, Yan Chen, Hongwen Li, Changkai Hou, Bangyue Wang, Yan Zhao, Xiangdong Wang, Gangfeng Yin, Xinyu Yang

**Affiliations:** ^1^ Department of Neurosurgery Tianjin Medical University General Hospital Tianjin China; ^2^ Department of Neurosurgery Tianjin Huanhu Hospital Tianjin China; ^3^ Department of Neurosurgery Second Hospital of Hebei Medical University Hebei China; ^4^ Department of Geriatrics Liaocheng People's Hospital Shandong China; ^5^ Department of Neurosurgery Xuanwu Hospital Capital Medical University Beijing China; ^6^ Department of Neurosurgery Changzhi Medical College Affiliated Heji Hospital Shanxi China; ^7^ Department of Neurosurgery Cangzhou Central Hospital Hebei China

**Keywords:** cerebrovascular diseases, epidemiology, neurosurgery, stroke

## Abstract

**Objective:**

The study evaluated the effectiveness and safety of single‐stage versus multistage endovascular treatment in subarachnoid hemorrhage patients with Mirror Aneurysms.

**Materials and Methods:**

Our research team performed a prospective study, focusing on the radiographic and clinical data of patients diagnosed with subarachnoid hemorrhage, specifically those who presented with Mirror Aneurysms upon admission to our institutions. According to the different endovascular treatment stages, these patients were grouped into the multistage cohort and the single‐stage cohort.

**Result:**

A total of 216 aneurysms were identified among the 108 patients, with every patient having one ruptured aneurysm. The duration of follow‐up was 2 years in both groups. In the single‐stage cohort, all 114 aneurysms in 57 patients were managed during a single session. During the 2‐year follow‐up, it was observed that 49 patients achieved a modified Rankin Scale score ≤2. Five complications were encountered, including cerebral vasospasm in three patients, cerebral hemorrhage in one patient, and thromboembolism in one patient. In the multistage cohort, only the ruptured aneurysm (amounting to 51 in total) received treatment at the initial occurrence, while the remaining 51 aneurysms were addressed subsequently. Throughout the 2‐year follow‐up period, 46 subjects exhibited a modified Rankin scale score ≤2. Overall, four complications were documented, including cerebral vasospasm in two patients, a subarachnoid hemorrhage in one patient, and thromboembolism in one patient.

**Conclusion:**

The safety and effectiveness of both endovascular treatment groups have been verified for patients with Mirror Aneurysms suffering from subarachnoid hemorrhage. If feasible, single‐stage embolization should be considered a viable treatment option for these patients.

## Graphical abstract

This study eaxmined the effectiveness and safetybetween single‐stage and multi‐stage endovascular treatment in subarachnoid haemorrhage patients with mirror aneurysms over the 2‐year follow‐up. In terms of complication rate and MRS score, there was no difference between single and multiple intravascular treatment of mirror intracranial aneurysms in patients with subarachnoid hemorrhage. If feasible, single‐stage coil embolization should be considered as an excellent means of treating mirror aneurysms in subarachnoid hemorrhage patients.

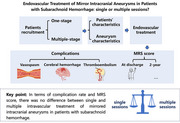

## 1 Introduction

Mirror Aneurysms (MirAns), colloquially referred to as “twin” aneurysms, represent a subgroup of multiple aneurysms, accounting for 2−12% of intracranial aneurysms (IAs) (Choi et al. 2018; Meissner et al. 2012). Previous publications have presented various terms for MirAns alongside distinct definitions (Liu et al. 2019; Wang et al. 2014; Casimiro et al. 2004; Mehrotra et al.^2016^). In this current research, MirAns was specifically characterized as “IAs that occur symmetrically and simultaneously on the intracranial vessels.”

IAs represent the predominant cause of subarachnoid hemorrhage (SAH), constituting approximately 3–10% of all strokes and exhibiting a mortality rate of 50% (Choi et al. 2018; Go et al. 2013; Meissner et al. 2012; Tawk et al. 2021). Patients presenting with SAH who remain untreated at the time of initial presentation exhibit a 1‐year mortality rate of up to 65% (Korja et al. 2017). Conversely, with appropriate therapeutic interventions, this mortality rate significantly declines to 18% (Lantigua et al. 2015).

Determining the optimal treatment strategy necessitates a comprehensive assessment encompassing the patient's clinical status and coexisting conditions, the site and the morphological features of the ruptured aneurysm (Claassen and Park 2022; Tawk et al. 2021). However, most previous clinical studies on MirAns in patients with SAH were few case reports or some single‐center studies, with a small sample size and poor representation (Casimiro et al. 2004; Meissner et al. 2012; Yamada et al. 2000). Currently, there is no universally accepted procedure for managing SAH patients with MirAns. The important clinical decision in managing patients of this nature revolves around the choice between an initial strategy focused on treating the ruptured aneurysm first, followed by subsequent treatment of the other aneurysms at a later session, or adopting a single‐stage treatment to all aneurysms simultaneously (Choi et al. 2018; Zhang et al. 2023). A multicenter, prospective, and observational cohort study evaluated the efficacy and safety of single‐session and multisession endovascular interventions for managing MirAns patients with SAH.

## 2 Materials and Methods

### 2.1 Patients

Patients with SAH were enrolled from the Chinese Multi‐Centre Cerebral Aneurysm Database (CMAD). The CMAD has been registered as a large multicenter, prospective, observational databank since 2016. Our team sequentially enrolled adult patients who received their initial diagnosis of SAH between January 1, 2017, and December 31, 2020. We conducted a comprehensive review of patient charts from a multicenter collaborative database comprising 5837 individuals diagnosed with SAH across 12 participating centers in China. These centers are located in Tianjin, Shandong, Shanxi and Hebei provinces.

The patient's data for the CMAD study were meticulously extracted from their medical records. The selection of the treatment strategy, whether to choose surgical treatment or endovascular treatment, was determined by the same multidisciplinary team comprising neurosurgeons and neurointerventionalists. The research protocol adhered to the principles outlined in the Declaration of Helsinki and received ethical clearance from the Ethics Committee of Tianjin Medical University General Hospital (No. IRB2022‐YX‐175‐01). Written informed consent was obtained from all patients.

The baseline characteristics of patients were recorded (including age, sex, medical history of hypertension, diabetes, ischemic stroke, intracerebral hemorrhage (ICH), smoking, drinking, Hunt‐Hess (HH) grade, time from onset to admission) and location of aneurysm.

### 2.2 Inclusion Criteria

The inclusion criteria were as follows:

1. The age of patients was ≥18 years.
2. Patients with a definite SAH, proven by brain computed tomography (CT).
3. Patients who were diagnosed with MirAns through the utilization of digital subtraction angiography (DSA).
4. All patients in this research who received endovascular treatment for aneurysm embolization.

### 2.3 Exclusion Criteria

The exclusion criteria were as follows:

1. The SAH did not stem from a ruptured aneurysm.
2. History of SAH or previous repair of IAs.
3. Patients afflicted with alternative cerebrovascular disorders, including Moyamoya disease and cerebrovascular anomalies.
4. Patients who opted for selective management of ruptured aneurysms while adopting a conservative approach towards unruptured aneurysms.

### 2.4 Endovascular Technique

In the research department, all patients receive pretreatment CT scans, and intravenous nimodipine is consistently administered at a rate of 2 mg/h to patients in order to prevent the appearance of delayed cerebral ischemia.

An appropriate therapeutic approach hinges on the patient's clinical status, concurrent medical comorbidities, vascular architecture, and specific aneurysm features. In individuals undergoing stent placement, preprocedural administration of aspirin (300 mg) and clopidogrel (300 mg) is initiated ≥30 min prior to the intervention. The endovascular treatment is performed under general anesthesia. During general anesthesia administration, experienced anesthetists intravenously administered anesthetic drugs to patients. Briefly, the anesthetic drugs included muscular relaxants (cisatracurium or rocuronium), analgesics (remifentanil, fentanyl or sufentanil) and sedatives (propofol or dexmedetomidine). The selection of these anesthetic drugs was determined by the specific anesthesia requirements, with the dosage of each drug administered tailored to the patient's body weight. During the procedure, meticulous regulation of blood pressure is implemented. In cases where the ruptured aneurysm is targeted solely, it is recommended that treatment of the other aneurysms be deferred until the patient has recovered, after a minimum of 1 month following discharge from the hospital.

The endovascular treatment strategy was made by neurosurgical teams, considering the neurovascular experience, the patient's current state, and the morphological characteristics of aneurysms. The strategy is as outlined below (Society of Neurosurgery of Chinese Medical Association, Society of Cerebrovascular Surgery of Chinese Stroke Association, National Center for Neurological Disorders, National Clinical Research Center for Neurological Diseases 2024): (1) Treating all aneurysms concurrently when there are challenges in precisely identifying the responsible aneurysm or when managing a ruptured aneurysm could impact the unruptured aneurysm. (2) If the morphological and hemodynamic analysis presents a heightened risk of rupture for all aneurysms, we will adopt one‐stage treatment approach. (3) In the remaining instances, the treatment approach is determined by the doctor, considering the patient's condition, the attributes of the aneurysm, and the clinical expertise of the doctors. Participants were categorized according to the treatment strategy: one‐staged treatment and multiple‐stage treatment. In the one‐staged cohort, all IAs were dealt with embolization in a session. In the multiple‐stage cohort, only the ruptured IAs were dealt with embolization at the time of initial assessment, and the other IAs were recommended delayed treatment.

### 2.5 Clinical Evaluation

Our team uses the CMAD to identify individuals who met this study criteria and then contact those individuals for follow up. After a 2‐year discharge, follow‐up is conducted via telephone, during which all treatment and follow‐up complications are documented and subsequently evaluated. The outcome is assessed utilizing the modified Rankin scale (mRS), wherein a score of 0–2 is regarded as advantageous, while a score of 3–6 is deemed disadvantageous.

### 2.6 Statistical Analysis

Statistical analyses were conducted using SPSS software version 22.6 (Armonk, NY, USA). Normally distributed continuous data were presented as mean ± standard deviation (*M*±SD), while nonnormally distributed continuous data were presented as median. Categorical data were expressed as numbers with percentages and compared using the chi‐square test. Statistical significance was set at a *p*‐value of less than 0.05.

We checked the inter‐ and intrarater validity of the outcome measure before submission. Interrater validity: We computed the Kappa coefficient for ratings by two independent raters on the same data set to assess interrater agreement. The results indicated a Kappa coefficient of 0.85, indicating a high level of agreement among raters. This means this research is at the high level of interrater agreement. Intrarater validity: We invited one rater to assess the same data set twice at different time points and calculated the Pearson's correlation coefficient between these two assessments. The test‐retest reliability correlation coefficient was found to be 0.95, indicating a high level of stability and consistency in ratings across different time points. In conclusion, by using statistical methods such as the Kappa coefficient and test‐retest reliability, we thoroughly examined the inter‐ and intrarater validity of the outcome measure.

## 3 Results

**FIGURE 1:**
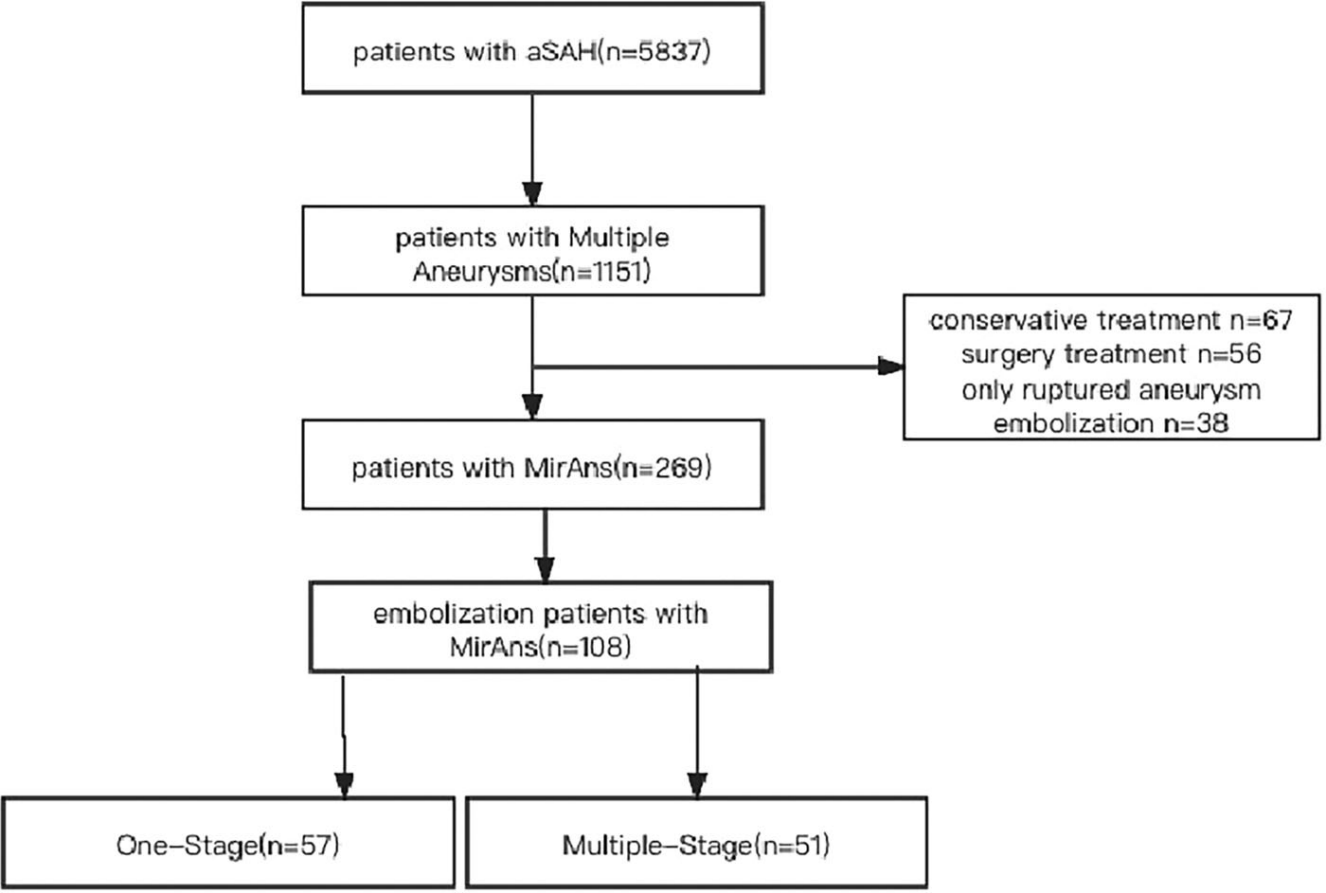
Flowchart of the study population.

From January 2017 to December 2020, a total of 5837 patients with SAH were admitted to the CMAD, of which 269 had MirAns in Patients with SAH accounting for 4.61%. Of the 269 SAH patients with MirAns, 161 patients were not including this study for they adopt conservative treatment (*n* = 67) or surgery treatment (*n* = 35) or hybrid treatment (*n* = 21) or only ruptured aneurysm embolization (*n* = 38). Eventually, MirAns in 108 Patients with SAH were eligible for inclusion in this 2‐year follow‐up, multicenter, prospective, observational study, and the flow chart is present in Figure 1.

Table 1 provides a comprehensive summary of the patient and aneurysm characteristics. The study population consisted of 108 patients, collectively harboring 216 aneurysms. Following the treatment, the Raymond–Roy classification was distributed as complete embolization (158 aneurysms), neck remnant (48 aneurysms), and sac remnant (10 aneurysms). According to Hunt‐Hess grading, the initial categorization ranged from I to III, with 46 cases (80.7%) in the one‐stage cohort and 52 cases (82.3%) in the multiple‐stage cohort. A combined total of 51 aneurysms were subjected to coil embolization as the sole intervention, out of which 21 were aneurysms that had ruptured. In the single‐stage cohort, the rate of stent utilization for the responsible aneurysm was 42.1%, which was higher than the rate of 41.2% observed in the staged treatment group. No statistically significant variances were observed about gender, age, hypertension, diabetes, ischemic stroke, intracerebral hemorrhage (ICH), smoking, alcohol consumption, aneurysm location, Hunt and Hess (H‐H) grade, and stent assistance between the one‐staged treatment group and the group undergoing treatment for ruptured aneurysms.

**TABLE 1:** Patients' characteristics and aneurysm characteristics.

	One stage (*n* = 57)	Multiple stage (*n* = 51)	All	*p* Value
Gender			108	
Male	18 (31.6%)	23 (45.1%)	41 (38.0%))	0.092
Female	39 (68.4%)	28(54.9%)	67 (62.0%)	
Age, yeas (*M* ± SD)	62.2±7.9	59.7±8.5		0.364
Medical History				
Hypertension	27 (47.4%)	29 (56.9%)	58 (53.7%)	0.276
Diabetes	8 (14.0%)	6 (11.8%)	14 (13.0%)	0.119
Ischemic stroke	5(8.8%)	7(13.7%)	12 (22/2%)	0.174
Intracerebral hemorrhage	3 (5.3%)	2 (3.9%)	5 (7.4%)	0.547
Drinking	10 (17.5%)	8 (15.6%)	18 (16.7%)	0.331
Smoking	8 (14.0%)	9 (17.6%)	17 (15.7%)	0.215
Hunt‐Hess score	57	51		
I–III	46 (80.7%)	42 (82.3%)	88 (81.5%)	0.694
IV–V	11 (19.3%)	9 (17.7%)	20 (18.5%)	
No. of aneurysm treated	114	102	216	
Location of aneurysms(ruptured/unruptured)	57/57	51/51	108/108	
Anterior cerebral artery	4 (7.0%)/1 (1.7%)	4 (7.8%)/1 (2.0%)	8 (7.4%)/2 (1.9%)	0.197
Anterior communicating artery	6 (10.5%)/3 (5.3%)	5 (9.8%)/2 (3.9%)	11 (10.2%)/5 (4.6%)	0.897
Internal carotid artery	7 (12.3%)/11 (19.3%)	5 (9.8%)/10 (19.8%)	12 (11.1%)/21 (19.4%)	0.672
Middle cerebral artery	13 (6.5%)/10 (2.9%)	10 (6.5%)/7 (13.7%)	23 (21.3%)/17 (15.7%)	0.374
Posterior communicating artery	23 (40.4%)/29 (50.9%)	25 (49.0%)/28 (54.5%)	48 (44.4%)/57 (52.8%)	0.849
Basilar artery	3 (5.3%)/3 (5.3%)	2 (3.9%)//2 (3.9%)	5 (4.6%)/5 (4.6%)	0.723
Posterior cerebral artery	1 (1.8%)/0 (0%)	0 (0%)/1 (2.0%)	1 (0.1%)/1 (0.1%)	1.000
Stent assist (total aneurysms)	53/114	41/102	94/216	
Ruptured aneurysms	24/57 (42.1%)	21/51 (41.2%)	45/108 (41.6%)	0.489
Unruptured aneurysms	29/57	30/51		
Raymond‐Roy occlusion classification	114	102	216	
1	80 (70.2%)	78 (76.5%)	158 (73.1%)	0.978
2	29 (24.5%)	20 (19.6%)	49 (22.7%)	0.815
3	5 (5.3%)	4 (3.9%)	9 (4.2%)	0.933

### 3.1 Complications

Overall, nine complications were observed during the peri‐ and postprocedural follow‐up, with five cases (8.8%) occurring in the single‐stage cohort and 4 cases (7.8%) in the multiple‐stage cohort. No statistically significant disparities were identified in terms of complications between the cohort receiving one‐stage treatment and the cohort undergoing multiple‐stage treatment.

In the single‐stage cohort, intraprocedural cerebral vasospasm was observed in three individuals (5.7%). The etiology was SAH‐related vascular irritation in two cases and surgical manipulation in one case.

All patients exhibited vasospasm enhancements after administering nimodipine and retraction of the guiding catheter without any concurrent neurological impairments. Among the one‐stage treatment patients, one individual (1.8%) experienced cerebral hemorrhage, diagnosed explicitly as hypertensive cerebral hemorrhage, 1 year following the implementation of stent‐assisted embolization.

The patient demonstrated clinical improvement following the implementation of supportive care. Thromboembolic episodes were observed in one (1.8%) individual from the one‐stage treatment group. This patient experienced a visual field defect in the upper outer quadrant of the right eye, which showed no signs of amelioration despite administering intravenous tirofiban. An ophthalmological evaluation indicated the presence of embolization in a branch of the central retinal artery in this specific case. This patient underwent coil embolization as the sole treatment for the ruptured aneurysm, and stent‐assisted coil embolization was employed for the management of the unruptured aneurysm.

Within the cohort subjected to multiple‐stage treatment, four (7.8%) patients experienced intraprocedural cerebral vasospasm. The origin of this phenomenon was ascribed to SAH‐induced vascular irritation in one patient and surgical manipulation in another patient, with neither demonstrating any subsequent neurological impairments. A patient who underwent multistage therapy exhibited a recurrence of subarachnoid hemorrhage (Re‐SAH). This patient, who underwent stent‐assisted coil embolization for the ruptured aneurysm during the initial phase, did not follow our advice for immediate second‐stage intervention for the residual aneurysm. After the rupture of the untreated aneurysm, stent‐assisted coil embolization was implemented for its embolization. Subsequently, a patient within the single‐stage treatment cohort encountered thromboembolic episodes, resulting in the manifestation of impaired function in the right hand, which subsequently exhibited improvement after the administration of intravenous tirofiban.

### 3.2 Clinical Outcomes and Follow‐Up

Table 2 presents the data about the procedural and postprocedural follow‐up. 21 (19.4%) of 108 patients were known to have poor outcomes (mRS 3–6) at the time of discharge, while 12 (21.1%) of 57 allocated one‐stage treatment and 9 (17.7%) of 51 allocated multiple‐stage treatment. 

**TABLE 2:** The complications and follow‐up outcome of the patients.

	One stage (*n* = 57)	Multiple stage (*n* = 51)	*p* Value
Complications	5(8.8%)	4(7.8%)	0.571
Vasospasm	3 (5.3%)	2 (3.9%)	0.553
Cerebral hemorrhage	1 (1.8%)	1 (2.0%)	0.724
Thromboembolism	1 (1.8%)	1 (2.0%)	0.724
MRS score at discharge			
0–2	45 (78.9%)	42(82.3%)	0.398
3–6	12 (21.1%)	9(17.7%)	
MRS score at 2 years			
0–2	42(84.0%)	38(86.4%)	0.431
3–6	8(16.0%)	6(13.6%)	

With a follow‐up rate of 87.0% (*n* = 94) at 2 years, the rate of poor outcome was 16.0% (*n* = 8) in the group of one‐staged treatment and 13.6% (*n* = 6) in the group of multiple‐stage treatment. A total of 14 patients (remaining 13%) were lost to follow‐up due to incorrect telephone numbers. Seven patients in the one‐stage cohort were lost, while seven patients in the multiple‐stage cohort. The poor outcome rate of the one‐stage treatment group at discharge and 2‐year follow‐up is not higher than that of the multiple‐stage treatment group (*p* = 0.398, 0.431).

## 4 Discussion

MirAns occur in 2−12% of patients with IAs in the world (Choi et al. 2018; Meissner et al. 2012). Although they occur frequently, they have not been thoroughly researched. Previous reports have provided comprehensive descriptions of the spontaneous development of mirror aneurysms (Casimiro et al. 2004; Liu et al. 2019); however, there remains a dearth of thorough investigation into managing these lesions. According to the findings reported by Mehrotra et al. (2016), surgical clipping has demonstrated superior neurological outcomes in 17 cases of ruptured mirror aneurysms compared to multiple nonmirror aneurysms. This approach was associated with fewer intraoperative rupture, vasospasm, and infarction incidences.

Similarly, Wang et al. (2014) conducted a study involving a cohort of 43 patients with MirAns, the majority of whom underwent clipping (*n* = 39) rather than coil embolization (*n* = 4). Several case series reports have extensively described the management of mirror aneurysms, including clipping and coil embolization techniques (Baccin et al. 2006; Stamatopoulos et al. 2021; Wang et al. 2017). However, unlike the limited number of MirAns patients with SAH amassed by endovascular treatment in prior efforts, this study included a large sample of MirAns patients treated with SAH in China.

Historical endeavors to perform simultaneous surgical clipping of multiple intracranial aneurysms typically yielded unfavorable results, primarily attributable to extensive manipulation of cerebral arteries, the necessity for bilateral craniotomy, and prolonged retraction of brain tissue (Mizoi, Suzuki, and Yoshimoto 1989; Rinne et al. 1995). Nevertheless, recent advancements in microsurgical techniques have facilitated bilateral multipoint access through a unilateral approach (Hong and Wang 2009; Rodríguez‐Hernández, Gabarrós, and Lawton 2012; Wachter et al. 2011). However, the limited surgical exposure presents challenges in effectively managing intraoperative rupture, thereby increasing the susceptibility of the olfactory bulb rootlet to injury (Rodríguez‐Hernández et al., 2012). Furthermore, the presence of severe subarachnoid hemorrhage (SAH) accompanied by brain swelling and reduced subfrontal corridor, which hampers the feasibility of single‐stage clipping, serves as a contraindication (Brown 2010). In MirAns, the feasibility of dual clipping through a unilateral approach is further constrained by the considerable distance between the two lesions. Moreover, when compared to endovascular coiling, the utilization of bilateral surgical clipping (which necessitates bilateral craniotomy) is associated with increased invasiveness, potentially prolonged operative time, and heightened risk of excessive blood loss (Choi et al. 2018). Without substantial technical limitations, our institutions favor single‐stage embolization over single‐stage surgical clipping.

In patients with SAH and MirAns, prompt and proactive treatment of the ruptured aneurysm is imperative to prevent subsequent ruptures (Choi et al. 2018; Zhang et al. 2023). However, the management of unruptured aneurysms remains a subject of controversy (Liu et al. 2019; Wang et al. 2014). It has been postulated that in SAH patients with MirAns, there is an elevated risk of surgical complications associated with the simultaneous treatment of unruptured aneurysms in a single‐stage procedure (Hadjiathanasiou et al. 2020). Li et al. (2018) demonstrated the safety of one‐stage treatment irrespective of acute‐phase bleeding in MirAns. In our investigation, although the one‐stage cohort exhibited a higher incidence of complications than the staged cohort, no statistically significant discrepancy was detected in these two cohorts (*p* = 0.318).

Given that the yearly incidence of rupture for unruptured intracranial aneurysms stands at 1.9%, certain authorities have proposed that comprehensive single‐stage treatment of all IAs could not only reduce the probability of aneurysm rupture but also result in cost savings for patients in terms of treatment expenditures (Rinkel et al. 1998). Additionally, Hino et al. conducted a therapeutic intervention on a total of 76 cases presenting with multiple aneurysms, revealing that out of the six patients who were incorrectly diagnosed as having ruptured aneurysms, four experienced instances of postoperative rebleeding (Hino et al. 2000). These findings offer supporting evidence for the implementation of single‐stage embolization as a preventive strategy to mitigate the complications resulting from unruptured aneurysm rupture and misdiagnosis of ruptured aneurysms in MirAns cases. In our research, a single patient did not receive timely treatment for an unruptured aneurysm and experienced a recurrent bleeding episode during the follow‐up period.

The modified Rankin Scale (mRS) is a commonly used scale for measuring the degree of disability or dependence in the daily activities of people who have suffered a stroke or other causes of neurological disability (Haggag and Clinimetrics 2022). It has become the most widely used clinical outcome measure for aneurysmal SAH clinical trials (Tjerkstra et al. 2024). Traditionally, it has been administered through face‐to‐face interviews or paper‐based questionnaires. However, with advances in technology, it is now feasible to administer the mRS using mobile applications or online platforms. Several prior studies (An et al. 2024; Wang et al. 2023; Yang et al. 2023) have investigated the reliability and validity of rating the mRS remotely through phone or video calls.

In the cases of MirAns patients with SAH, both one‐stage and multiple‐staged cohorts have their strengths. In cases of SAH in patients with MirAns, both single‐stage and multistage treatment approaches demonstrate their respective merits. The one‐stage cohort exhibited several advantages. In this cohort that underwent one‐stage treatment, several benefits were observed. Firstly, the risk of rupture in untreated unruptured aneurysms was effectively avoided (Rinkel et al. 1998). Additionally, the patient's economic impact significantly decreased. One‐stage procedures have the potential for significant cost savings (Choi et al. 2018). Xavier et al. (2012) reported mean cost of hospital charges in the one‐stage cohort was $20,948, which was lower than that ($38,263) for the multiple‐stage cohort. Savings encompass the expenses related to administering general anesthesia, utilizing the angiographic suite, staying in the intensive care unit and regular ward bed. Compared to the multiple‐stage cohort, the one‐stage cohort eliminates repeated groin punctures and general anesthesia. Furthermore, patient concerns and anxieties related to the potential rupture of unruptured aneurysms are alleviated (Tawk et al. 2021). In this investigation, the one‐stage cohort reported a total of five complications. Notably, one patient encountered thromboembolism in a branch of the central retinal artery. This unfavorable occurrence can be attributed to the duration of the procedure and the need for continuous saline infusion through the introducer catheter.

Multiple‐stage cohorts can also offer certain positive aspects. Firstly, it prevents dual antiplatelet therapy during the acute post‐SAH (Zhang et al. 2023). In the case of the staged treatment group, our preference is to perform coiling alone as the first‐stage procedure for embolizing the ruptured aneurysm, thereby circumventing the need for stents (except for some specific types of aneurysms). A significantly elevated occurrence of bleeding and ischemic complications has been documented in patients with SAH who undergo stent‐assisted coil embolization (Nagahama et al. 2018). Second, a singular emphasis on addressing the ruptured aneurysm reduces the duration of the procedure. Prolonged procedural time is linked to an increased frequency of ischemic and hemorrhagic complications. Thirdly, patients in critical condition frequently encounter challenges in tolerating protracted procedures (Zhang et al. 2023). Choi et al. (2018) conducted a clinical investigation involving 146 patients (totaling 292 MirAns) who underwent one‐stage treatment. The immediate posttreatment classification according to the Raymond‐Roy scale was as follows: complete embolization (31 aneurysms), neck remnant (94 aneurysms), and sac remnant (3 aneurysms). The incidence of complications (3 out of 146, 2.1%) and the achievement of complete occlusion postprocedure (31 out of 146, 21.2%) were comparable to our findings. In our study, the complications (5 of 57, 8.8%) and postprocedural complete occlusion results (80 of 114, 70.2%) were within the single‐stage treatment group. The complications are lower in Choi et al. than in our study. However, our study's postprocedural complete occlusion results are higher than those of Choi et al.

**FIGURE 2:**
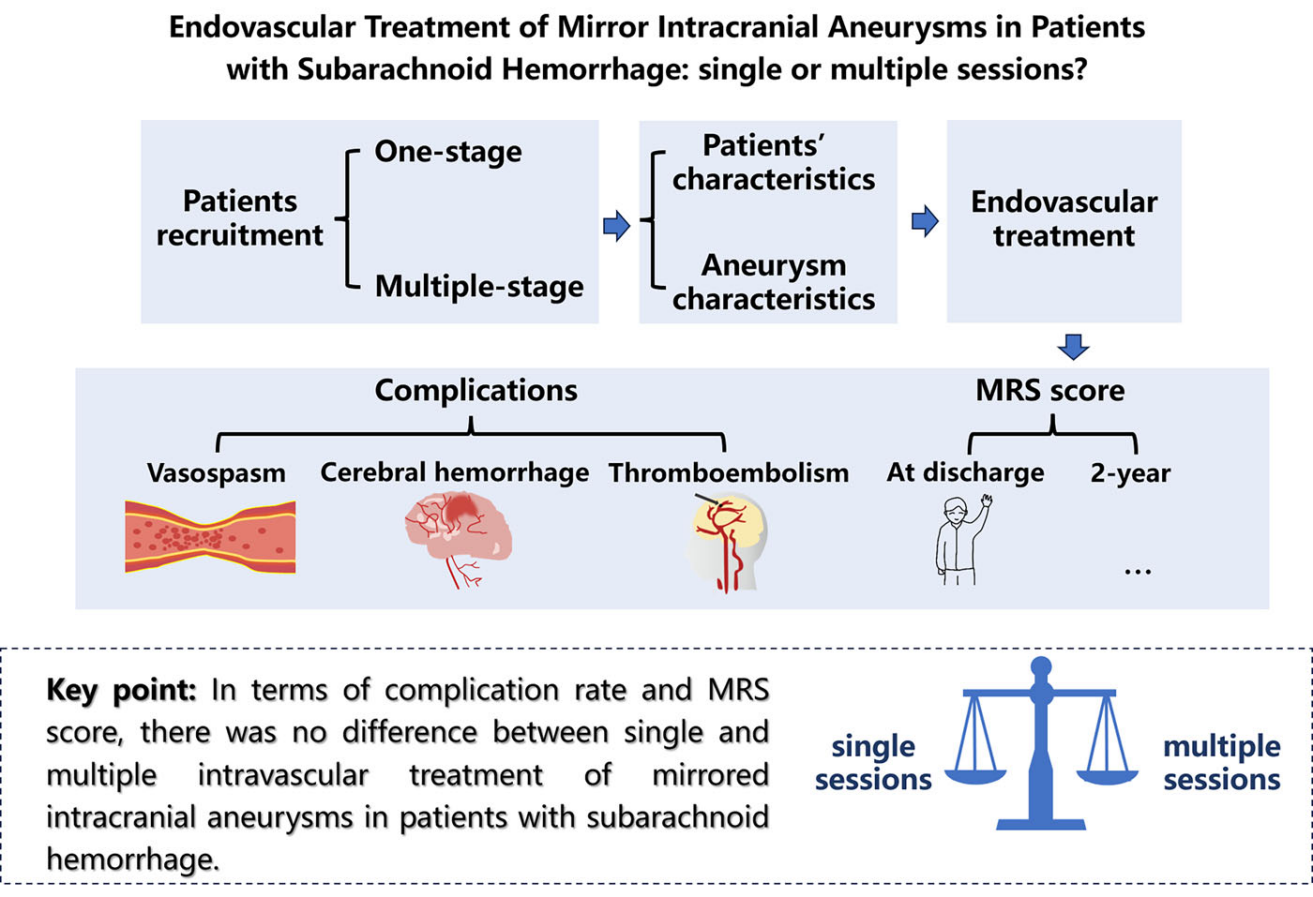
Graphical abstract and graphical text.

In our study, the complications rate is higher in the group of one‐stage treatment than in the multiple‐stage cohort, but there was no statistical difference between the two cohorts (*p* = 0.318). The mRS of patients at discharge and 2 years after discharge in the multiple‐stage treatment group is higher than the group, but there was no statistical difference between the two cohorts (*p* = 0.398, 0.431). The complications and mRS score of these cohorts is shown in Figure 2. Therefore, single‐stage embolization is proposed as a viable option for the MirAns in patients with SAH.

## 5 Conclusion

SAH in patients with mirror aneurysms presents a challenging clinical scenario. Identifying and treating the ruptured aneurysm is crucial and should be approached proactively. Single‐stage and multiple‐stage endovascular treatments have demonstrated safety and efficacy in patients with aneurysmal SAH associated with mirror aneurysms. In cases where it is possible, the utilization of single‐stage embolization should be contemplated as an exceptional approach for managing (MirAns in patients suffering from SAH. Nonetheless, it is imperative to include a larger sample size to establish both groups' treatment efficacy.

### 5.1 Limitation

Several limitations are evident in our study. Firstly, we have not detected relevant difference between the treatment strategies due to the small sample size of patients. A larger volume of cohort is what we plan to further study in the future. Second, the extended treatment duration may have led to changes in treatment approaches due to the advancements in endovascular technique and embolization material. Third, the sequential enrollment of patients does not significantly mitigate selection bias. Next, our team will carry out a Randomized Controlled Trial on MirAns in SAH patients to eliminate this bias. Lastly, the lack of mRS at baseline is another limitation in this study. Moreover, the mRS scores at the 3‐year follow‐up are currently being gathered, and the change in mRS will be analyzed in subsequent evaluations as well.

## Author Contributions

**Yunfei Song**: conceptualization, methodology, investigation, writing—original draft. **Guoqiang Song**: conceptualization. **Guijing Liu**: software. **Limei Mao**: data curation. **Xiuhu An**: investigation. **Chao Peng**: validation. **Jian Li**: formal analysis. **Yan Chen**: supervision. **Hongwen Li**: formal analysis. **Changkai Hou**: writing—original draft. **Bangyue Wang**: writing—review and editing. **Yan Zhao**: writing—review and editing. **Xiangdong Wang**: resources. **Gangfeng Yin**: project administration. **Xinyu Yang**: project administration, resources. All authors contributed to the article and approved the submitted version.

## Ethics Statement

The study was conducted in accordance with the Declaration of Helsinki and was approved by the Ethics Committee of Tianjin Medical University General Hospital (No. IRB2022‐YX‐175‐01).

## Conflicts of Interest

The authors declare that the research was conducted in the absence of any commercial or financial relationships that could be construed as a potential conflict of interest.

### Peer Review

The peer review history for this article is available at https://publons.com/publon/10.1002/brb3.70234.

## Data Availability

Due to ethical and privacy constraints, the data sets presented in this article are not openly accessible. Requests for data set access should be directed to the corresponding author.

## References

[brb370234-bib-0001] An, X. , J. Su , B. Duan , et al. 2024. “Clinical Characteristics and Outcomes in Patients With Ruptured Middle Cerebral Artery Aneurysms: A Multicenter Study in Northern China.” *Neurology and Therapy*. 10.1007/s40120-024-00673-y. Epub ahead of print. PMC11762049 39485598

[brb370234-bib-0002] Baccin, C. E. , T. Krings , H. Alvarez , A. Ozanne , and P. Lasjaunias . 2006. “Multiple Mirror‐Like Intracranial Aneurysms. Report of a Case and Review of the Literature.” Acta Neurochirurgica 148, no. 10: 1091–1095. 10.1007/s00701-006-0860-z. 16896548

[brb370234-bib-0003] Brown, R. D. 2010. “Unruptured Intracranial Aneurysms.” Seminars in Neurology 30, no. 5: 537–544. 10.1055/s-0030-1268858. 21207346

[brb370234-bib-0004] Casimiro, M. V. , A. W. McEvoy , L. D. Watkins , and N. D. Kitchen . 2004. “A Comparison of Risk Factors in the Etiology of Mirror and Nonmirror Multiple Intracranial Aneurysms.” Surgical Neurology 61, no. 6: 541–545. 10.1016/j.surneu.2003.08.016. 15165792

[brb370234-bib-0005] Choi, H. H. , Y. D. Cho , D. H. Yoo , et al. 2018. “Intracranial Mirror Aneurysms: Anatomic Characteristics and Treatment Options.” Korean Journal of Radiology 19, no. 5: 849–858. 10.3348/kjr.2018.19.5.849. 30174473 PMC6082764

[brb370234-bib-0006] Claassen, J. , and S. Park . 2022. “Spontaneous Subarachnoid Haemorrhage.” Lancet (London, England) 400, no. 10355: 846–862. 10.1016/S0140-6736(22)00938-2. 35985353 PMC9987649

[brb370234-bib-0007] Go, A. S. , D. Mozaffarian , V. L. Roger , et al. 2013. “Heart Disease and Stroke Statistics—2013 Update: A Report From the American Heart Association.” Circulation 127, no. 1: e6–e245. 10.1161/CIR.0b013e31828124ad. 23239837 PMC5408511

[brb370234-bib-0008] Hadjiathanasiou, A. , P. Schuss , S. Brandecker , et al. 2020. “Multiple Aneurysms in Subarachnoid Hemorrhage—Identification of the Ruptured Aneurysm, When the Bleeding Pattern Is Not Self‐Explanatory—Development of a Novel Prediction Score.” BMC Neurology 20, no. 1: 70. 10.1186/s12883-020-01655-x. 32113481 PMC7049209

[brb370234-bib-0009] Haggag, H. , and H. C. Clinimetrics . 2022. “Modified Rankin Scale (mRS).” Journal of Physiotherapy 68, no. 4: 281. 10.1016/j.jphys.2022.05.017. Epub 2022 Jun 15. 35715375

[brb370234-bib-0010] Hino, A. , M. Fujimoto , Y. Iwamoto , T. Yamaki , and T. Katsumori . 2000. “False Localization of Rupture Site in Patients With Multiple Cerebral Aneurysms and Subarachnoid Hemorrhage.” Neurosurgery 46, no. 4: 825–830. 10.1097/00006123-200004000-00011. 10764255

[brb370234-bib-0011] Hong, T. , and Y. Wang . 2009. “Unilateral Approach to Clip Bilateral Multiple Intracranial Aneurysms.” Surgical Neurology 72, no. Suppl 1: S23–S28. 10.1016/j.surneu.2007.12.031. 18514280

[brb370234-bib-0012] Korja, M. , R. Kivisaari , B. Rezai Jahromi , and H. Lehto . 2017. “Natural History of Ruptured but Untreated Intracranial Aneurysms.” Stroke; A Journal of Cerebral Circulation 48, no. 4: 1081–1084. 10.1161/STROKEAHA.116.015933. 28250196

[brb370234-bib-0013] Lantigua, H. , S. Ortega‐Gutierrez , J. M. Schmidt , et al. 2015. “Subarachnoid Hemorrhage: Who Dies, and Why?” Critical Care (London, England) 19, no. 1: 309. 10.1186/s13054-015-1036-0. 26330064 PMC4556224

[brb370234-bib-0014] Li, T. F. , S. F. Shui , X. W. Han , L. Yan , J. Ma , and D. Guo . 2018. “One‐Stage Endovascular Embolization for Multiple Intracranial Aneurysms.” Turkish Neurosurgery 28, no. 1: 43–47. 10.5137/1019-5149.JTN.18186-16.1. 27593847

[brb370234-bib-0015] Liu, H. J. , H. Zhou , D. L. Lu , et al. 2019. “Intracranial Mirror Aneurysm: Epidemiology, Rupture Risk, New Imaging, Controversies, and Treatment Strategies.” World Neurosurgery 127: 165–175. 10.1016/j.wneu.2019.03.275. 30954748

[brb370234-bib-0016] Mehrotra, A. , P. Sharma , K. K. Das , et al. 2016. “Mirror Aneurysms Among Multiple Aneurysms: Lesser of the Two Evils.” World Neurosurgery 92: 126–132. 10.1016/j.wneu.2016.04.124. 27185651

[brb370234-bib-0017] Meissner, I. , J. Torner , J. Huston 3rd , et al. 2012. “Mirror Aneurysms: A Reflection on Natural History.” Journal of Neurosurgery 116, no. 6: 1238–1241. 10.3171/2012.1.JNS11779. 22404675 PMC3914146

[brb370234-bib-0018] Mizoi, K. , J. Suzuki , and T. Yoshimoto . 1989. “Surgical Treatment of Multiple Aneurysms. Review of Experience With 372 Cases.” Acta Neurochirurgica 96, no. 1‐2: 8–14. 10.1007/BF01403489. 2929394

[brb370234-bib-0019] Nagahama, Y. , L. Allan , D. Nakagawa , et al. 2018. “Dual Antiplatelet Therapy in Aneurysmal Subarachnoid Hemorrhage: Association With Reduced Risk of Clinical Vasospasm and Delayed Cerebral Ischemia.” Journal of Neurosurgery 129, no. 3: 702–710. 10.3171/2017.5.JNS17831. 29099296

[brb370234-bib-0020] Rinkel, G. J. , M. Djibuti , A. Algra , and J. van Gijn . 1998. “Prevalence and Risk of Rupture of Intracranial Aneurysms: A Systematic Review.” Stroke; A Journal of Cerebral Circulation 29, no. 1: 251–256. 10.1161/01.str.29.1.251. 9445359

[brb370234-bib-0021] Rinne, J. , J. Hernesniemi , M. Niskanen , and M. Vapalahti . 1995. “Management Outcome for Multiple Intracranial Aneurysms.” Neurosurgery 36, no. 1: 31–38. 10.1227/00006123-199501000-00003. 7708165

[brb370234-bib-0022] Rodríguez‐Hernández, A. , A. Gabarrós , and M. T. Lawton . 2012. “Contralateral Clipping of Middle Cerebral Artery Aneurysms: Rationale, Indications, and Surgical Technique.” Neurosurgery 71, no. 1: Suppl Operative, 116–124. 10.1227/NEU.0b013e31824d8f66. 22307073

[brb370234-bib-0023] Society of Neurosurgery of Chinese Medical Association, Society of Cerebrovascular Surgery of Chinese Stroke Association, National Center for Neurological Disorders, National Clinical Research Center for Neurological Diseases . 2024. “Chinese Guideline for the Clinical Management of Patients With Ruptured Intracranial Aneurysms.” National Medical Journal of China 104, no. 21: 1940–1971. 10.3760/cma.j.cn112137-20240222-00374. 38825939

[brb370234-bib-0024] Stamatopoulos, T. , A. Mitsos , V. Panagiotopoulos , C. Tsonidis , A. Stamatopoulos , and P. P. Tsitsopoulos . 2021. “Demographic and Anatomical Comparison of Ruptured and Unruptured Intracranial Aneurysms: A Case Control Study.” Hippokratia 25, no. 3: 100–107. 36683906 PMC9851137

[brb370234-bib-0025] Tawk, R. G. , T. F. Hasan , C. E. D'Souza , J. B. Peel , and W. D. Freeman . 2021. “Diagnosis and Treatment of Unruptured Intracranial Aneurysms and Aneurysmal Subarachnoid Hemorrhage.” Mayo Clinic Proceedings 96, no. 7: 1970–2000. 10.1016/j.mayocp.2021.01.005. 33992453

[brb370234-bib-0026] Tjerkstra, M. A. , R. Post , M. R. Germans , et al. 2024. “ULTRA Trial Study Group. Ultra‐Early and Short‐Term Tranexamic Acid Treatment in Patients with Good‐ and Poor‐Grade Aneurysmal Subarachnoid Hemorrhage.” Neurology 102, no. 12: e209169. 10.1212/WNL.0000000000209169. Epub 2024 May 24. 38788175 PMC11226311

[brb370234-bib-0027] Wachter, D. , I. Kreitschmann‐Andermahr , J. M. Gilsbach , and V. Rohde . 2011. “Early Surgery of Multiple Versus Single Aneurysms After Subarachnoid Hemorrhage: an Increased Risk for Cerebral Vasospasm?.” Journal of Neurosurgery 114, no. 4: 935–941. 10.3171/2010.10.JNS10186. 21166569

[brb370234-bib-0028] Wang, B. Y. , C. Peng , H. S. Jiang , et al. 2023. “The Survival and Outcome of Older Patients With Primary Aneurysmal Subarachnoid Haemorrhage: a 2‐Year Follow‐Up, Multi‐Centre, Observational Study.” Age and Ageing 52, no. 11: afad202. 10.1093/ageing/afad202. 37979184

[brb370234-bib-0029] Wang, R. , D. Zhang , J. Zhao , S. Wang , Y. Zhao , and H. Niu . 2014. “A Comparative Study of 43 Patients With Mirror‐Like Intracranial Aneurysms: Risk Factors, Treatment, and Prognosis.” Neuropsychiatric Disease and Treatment 10: 2231–2237. 10.2147/NDT.S70515. 25429221 PMC4242700

[brb370234-bib-0030] Wang, W. X. , Z. Xue , L. Li , et al. 2017. “Treatment Strategies for Intracranial Mirror Aneurysms.” World Neurosurgery 100: 450–458. 10.1016/j.wneu.2017.01.049. 28131928

[brb370234-bib-0031] Xavier, A. R. , M. Rayes , P. Pandey , A. Tiwari , A. Kansara , and M. Guthikonda . 2012. “The Safety and Efficacy of Coiling Multiple Aneurysms in the Same Session.” Journal of NeuroInterventional Surgery 4, no. 1: 27–30. 10.1136/jnis.2009.001974. Epub 2011 Apr 14. 21990433

[brb370234-bib-0032] Yamada, K. , T. Nakahara , K. Kishida , T. Yano , K. Yamamoto , and Y. Ushio . 2000. “Multiple ‘Mirror’ Aneurysms Involving Intracavernous Carotid Arteries and Vertebral Arteries: Case Report.” Surgical Neurology 54, no. 5: 361–365. 10.1016/s0090-3019(00)00304-9. 11165612

[brb370234-bib-0033] Yang, Y. , B. Wang , X. Ge , et al. 2023. “Natural Course of Ruptured but Untreated Intracranial Aneurysms: A Multicenter 2‐Year Follow‐Up Study.” Stroke; A Journal of Cerebral Circulation 54, no. 8: 2087–2095. 10.1161/STROKEAHA.123.042530. Epub 2023 Jun 12. 37306018

[brb370234-bib-0034] Zhang, G. , W. Zhang , H. Chang , et al. 2023. “Endovascular Treatment of Multiple Intracranial Aneurysms in Patients With Subarachnoid Hemorrhage: One or Multiple Sessions?” Frontiers in Neurology 14: 1196725. 10.3389/fneur.2023.1196725. 37426436 PMC10325825

